# A plug-and-play, easy-to-manufacture fluidic accessory to significantly enhance the sensitivity of electrochemical immunoassays

**DOI:** 10.1038/s41598-024-64852-5

**Published:** 2024-06-19

**Authors:** Alexandra Dobrea, Nicole Hall, Stuart Milne, Damion K. Corrigan, Melanie Jimenez

**Affiliations:** 1 Biomedical Engineering Department, University of Strathclyde, Glasgow, G4 0NW UK; 2 James Watt School of Engineering, University of Glasgow, Glasgow, G12 8QQ UK; 3 Pure and Applied Chemistry Department, University of Strathclyde, Glasgow, G4 0NW UK

**Keywords:** Electrochemical immunosensor, Fluidics, Diagnostics, Assay systems, Diagnostic markers

## Abstract

Earlier access to patients’ biomarker status could transform disease management. However, gold-standard techniques such as enzyme-linked immunosorbent assays (ELISAs) are typically not deployed at the point-of-care due to their cumbersome instrumentation and complexity. Electrochemical immunosensors can be disruptive in this sector with their small size and lower cost but, without further modifications, the performance of these sensors in complex media (*e.g.*, blood) has been limited. This paper presents a low-cost fluidic accessory fabricated using widely accessible materials and processes for boosting sensor sensitivity through confinement of the detection media next to the electrode surface. Liquid confinement first highlighted a spontaneous reaction between the pseudoreference electrode and ELISA detection substrate 3,3’,5,5’-tetramethylbenzidine (TMB) that decreases the amount of oxTMB available for detection. Different strategies are investigated to limit this and maximize reliability. Next, flow cell integration during the signal amplification step of sensor preparation was shown to substantially enhance the detection of cytokine interleukin-6 (IL-6) with the best sensitivity boost recorded for fresh human plasma (x7 increase compared to x5.8 in purified serum and x5.5 in PBS). The flow cell requires no specialized equipment and can be seamlessly integrated with commercial sensors, making an ideal companion for electrochemical signal enhancement.

## Introduction

The early detection of biomarkers is instrumental in ensuring the best possible patient outcomes and minimizing the economic burden on healthcare systems through a personalized and preventative approach to disease. One of the gold standard techniques for quantifying biomarker levels in patient samples are enzyme linked immunosorbent assays (ELISAs); ELISAs exploit the high affinity between antibodies and their specific antigens to optically detect a biomolecular target, and are routinely used in care settings to detect a variety of conditions from bacterial^1,2^ or viral infections^3,4^, to autoimmune disorders^5^, and cancer^6^. Despite their wide use, the incurred high running costs and instrumentation footprint make standard ELISA approaches poorly suited for deployment at the point-of-care^7^.

Electrochemical immunosensors have been long hailed as the potential answer to this problem^8^. These sensors harness the reliability and selectivity of antibodies, small instrumentation size, low sample/reagent volumes, and the ability to be mass produced at low costs. Compared to their optical counterpart, electrochemical biosensors detect the antibody-antigen binding event through changes in the electrical profile of the medium *e.g.*, using amperometry, voltammetry, potentiometry or impedance spectroscopy. Another key strength of this sensing mechanism resides in its multiplexing capabilities^9^, which is critical for diseases that have a diverse biomolecular signature, and its strong point-of-care potential has already been demonstrated for *e.g.*, cardiovascular diseases^10^ and cancer^11^.

Translating ELISAs into an electrochemical format has however not been devoid of challenges. Without proper signal amplification strategies the sensitivity of these sensors has generally been quite low, particularly in complex media such as blood^12^. Consequently, a large body of research has been dedicated to improving the signal output using for instance surface modification with polymeric or metallic nanomaterials to increase the conductive and catalytic activity^13^, modulating the orientation of surface immobilized antibodies^12,14^ *e.g.*, through pH, click chemistry or covalent immobilization via SAM assembly (EDC/NHS), using nanobodies (shortened version of an antibody only including the active site), nanoparticles (AuNPs, IrOx etc.) and magnetic beads for increased surface area and conductivity^15,16^, or decreasing the electrode size into the micrometer realm^17^. Importantly however, all these modifications exponentially increase the production cost and manufacturing complexity of these sensors. Microfluidic strategies have also been proposed as a low-cost avenue to automate and increase the sensitivity and throughput of these sensors through minimization of environmental contamination, speeding up of reaction times (*e.g.*, through temperature control), decreasing the volume of sample and reagents, and gaining the ability to measure multiple biomarkers simultaneously through spatial separation of the fluid path^18–22^. Myriad microfluidic devices have been designed and presented mainly using polydimethylsiloxane (PDMS), glass etching, 3D printing, paper-based flow, CNC machining, thin film deposition and digital microfluidics^21,23–25^. While these manufacturing methods all have their merits and the choice of material and fabrication process is highly dependent on the nature of the final application, they still present some substantial limitations from a user adoption perspective in electrochemical biosensor development. PDMS devices for instance require an involved and somewhat lengthy fabrication process^26^ (mold design and production, degassing, curing, mold detachment, etc.), tend to swell over time when exposed to certain reagents and are prone to protein absorption^27^. 3D printed devices produced using stereolithography or fused-deposition techniques require post-treatment and curation to ensure that the photocuring agents do not foul the electrochemical transducer, and the surface finish is sufficiently smooth to not interfere with the flow or create air pockets^28^. Thin film deposition of microfluidic channels, while ideal for a final product, typically rely on high volume production methods and thus have limited prototyping flexibility and high start-up costs—the same applying to CNC machining^29^. Digital microfluidics have high power requirements and need complex engineering to set up, and thus might not be suitable or accessible for everyday research^23^.

While the clear benefits of microfluidic integration in electrochemical sensing have been previously demonstrated, microfluidic use in the community remains scarce, with only 4% of research outputs on electrochemical biosensors having a microfluidic element (Web of Science, 2020–2024).To improve user compliance, integration of a microfluidic element should have minimal disruption to the sensor testing workflow and should be easy to manufacture in bulk, even by non-microfluidic experts. Asri et al.^30^ recently reported for instance a simple and accessible way to manufacture both microfluidic devices and electrodes for electrochemical biosensors using consumer grade materials (gold leaf, silver ink pen, double sided tape, photocopier transparency film and heat and bond film) and processes (lamination, electronic craft cutting) for glucose sensing. Easier fluidic manufacturing could be achieved by working with existing, commercial electrodes and using consumer grade processes such as laser cutting, which is widely available within community-based ‘makerspaces’ such as the FabLab network (www.fabfoundation.org)^26^.

In this study, we explore this avenue and propose a new approach based on PMMA laser cutting for easy-to-manufacture and easy-to-assemble fluidic chambers that can improve the sensitivity of commercial electrodes. This approach is versatile, easily adaptable to different electrode designs, and can improve signals even in the absence of pumps to drive the flow - which can facilitate adoption by non-microfluidic experts. The principle is demonstrated here for an ELISA-based electrochemical detection of IL-6, an inflammatory cytokine produced by the immune system in response to an immunological challenge such as injury or infection. With relevance in a range of conditions such as sepsis, COVID-19, cancer or Alzheimer’s, IL-6 has garnered a high level of interest in the electrochemical biosensor community^31^. Using the proposed fluidic chamber, which is only added during the final step of electrochemical sensing to minimize interference, we demonstrate an increase in sensitivity (defined here as the change in current over the linear portion of the sensing range) of 5.5-fold in buffer, 5.8-fold in 10% v/v serum and 7-fold in neat, freshly extracted human plasma. This work consequently presents a rapid, low-cost and minimally invasive approach to enhance the signal of electrochemical sensors that can further enable deployment for point-of-care testing. The approach presented is unique in that in was specifically developed to facilitate uptake by the electrochemical biosensor community and we believe it can break down the barriers associated with microfluidic integration in these systems.

## Results and discussion

### Reliable cleaning procedures of commercial electrodes are critical for reproducible electrochemical sensing

**Figure 1:**
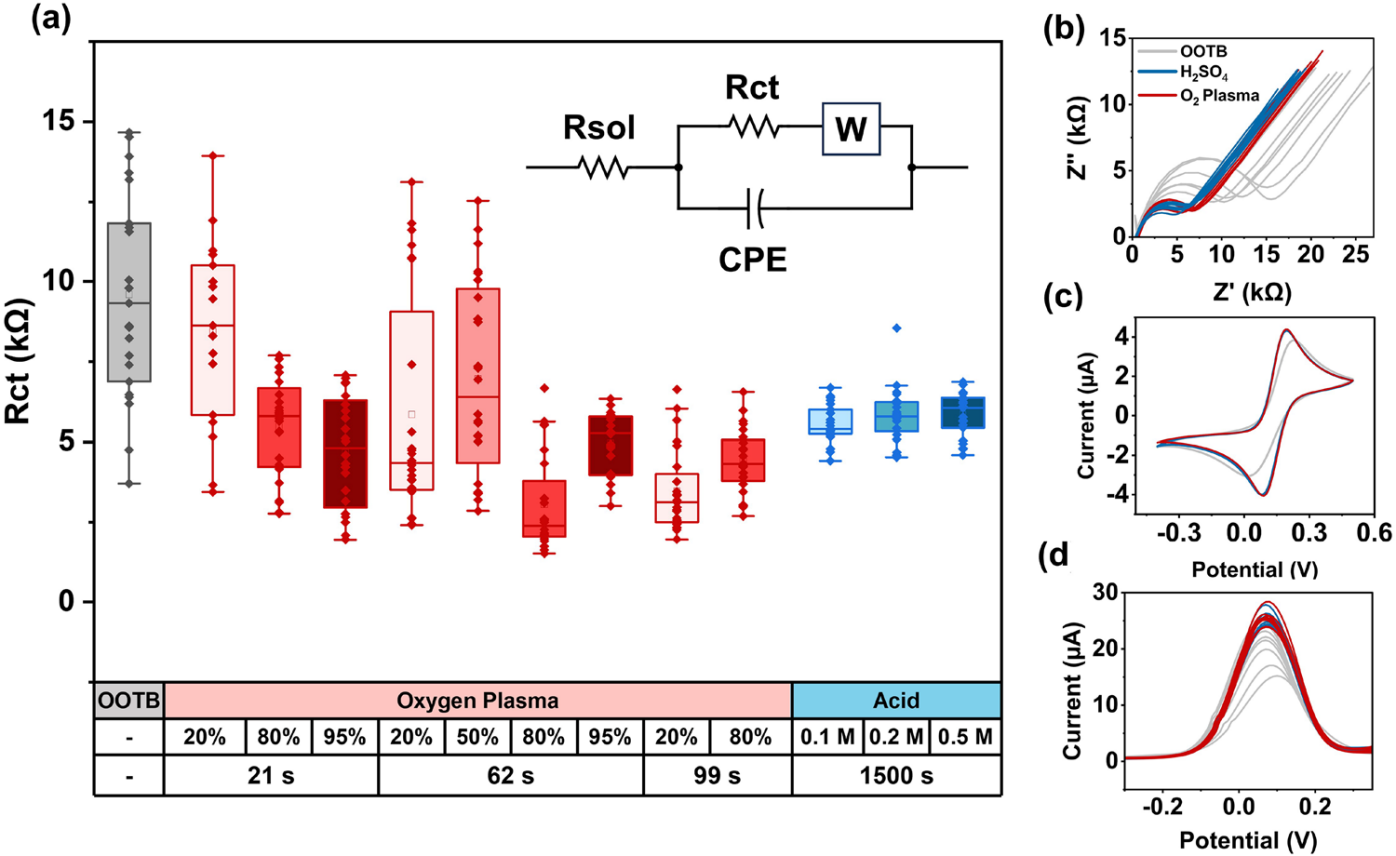
Comparison of the efficacy of oxygen plasma and sulfuric acid voltage cycling in removing contaminants from the gold electrode surface. (**a**) The charge transfer resistance (Rct) of the surface of commercial electrodes is measured through impedance spectroscopy without treatment (out of the box electrodes, OOTB), with plasma treatment at different powers (% of 200 W) and exposure times, and different sulfuric acid molarities. Data was fitted using Randles equivalent circuit (inset) to obtain the Rct values (Rsol = solution resistance, CPE = constant phase element, W = Warburg impedance). Datapoints represent different screen-printed electrode platforms with 8 working electrodes (n = 24 except 20% power 21s which had n = 16). Each box delineates the interquartile range (IQR), the straight line denotes the median, the hollow square represents the mean and whiskers denote the range within 1.5xIQR. (**b**) Examples of Nyquist plot, (**c**) Cyclic voltammogram and (**d**) Differential pulse voltammogram of an electrode without treatment (OOTB, grey) in comparison with one treated with the optimal plasma (95%, 62 s, red) and acid cycling cleaning (0.1 M, blue) protocols identified (n = 8). The response was measured using 2 mM ferri-ferrocyanide in 1xPBS.

Gold (Au) is an excellent material for electrochemical sensing due to its high stability, chemical inertness and high electrical conductivity which yields highly sensitive sensors with low background noise. As presented in Fig. 1, commercial electrodes out of the box (OOTB) can however have contaminants on their surface, introduced either during the manufacturing process or during storage and transport, which can lead to a large variability in the electrode’s response. To prevent this, cleaning procedures must be deployed to remove these impurities; this is particularly important in sensors employing non-covalently attached antibodies, where the sensitivity may already be lower due to the non-uniform orientation of the attached antibodies and consequent blocking of some of the active binding sites^14^. Sulfuric acid voltage cycling and plasma etching are two popular non-destructive techniques for electrode cleaning^32–35^ that were tested in this work. Both techniques work by removing the top layer of gold and any attached contaminants without substantially altering the surface morphology^36^.

Evaluation of the electrode surface using cyclic voltammetry, differential pulse voltammetry and impedance spectroscopy in a standard redox media (2 mM $Fe[CN]_{6}^{3-} + Fe[CN]_{6}^{4-}$ in 1xPBS) shows that treatment with both plasma and acid cleaning significantly (*p* < 0.01, Student’s t-test) reduced the charge transfer resistance (Rct) from 9593.8 ± 3189.1 $\Omega$ to 4961.3 ± 1025.7 $\Omega$ (62s 95%) and 5569.7 ± 599.6 $\Omega$ (0.1 M) respectively, suggesting improved accessibility of charges to the electrode surface (Fig. 1a,b). The peak separation voltage of the cyclic voltammogram ($\Delta$Ep) was also reduced from 201.1 ± 35.0 to 107.9 ± 6.6 and 106.6 ± 6.6 mV respectively (Fig. 1c), indicating improved reaction reversibility and electrode cleanliness. In an ideal system, where the reaction is chemically and electrochemically reversible, $\Delta$Ep should be $\sim$57 mV (at 25 $^{\circ }$C)^37^; deviation from this value could be attributed to imperfections in the electrode surface or the presence of trace amounts of contaminants remaining on the surface after cleaning.

Although plasma cleaning is a faster process ($\sim$ minutes to clean multiple electrodes) and is thought to have a more uniform and reproducible effect on the electrode surface compared to liquid solvents^38,39^, acid cleaning yielded lower coefficients of variability (CoVs) compared to any of the plasma settings investigated (Table S1). This could be due to the formation of gold oxides on the topmost layer of gold following $O_{2}$ plasma exposure behaving in a contaminant-like fashion^35^ or acting as a protective layer which consequently decreased the cleaning efficacy. The best settings identified for plasma in terms of CoV was a 95% power (or 190 W) for 62 s, but this already started damaging the electrode connector pads which began to shrink and fold in, likely because of overheating in the areas where the largest amount of gold was present. In terms of differential pulse voltammetry (DPV) peak currents (Ipeak)—and as shown in Fig. 1d—both plasma and acid treatment substantially reduced the variability of the sensors. Although plasma etching typically produced higher currents compared to acid (Fig. S2), the variability between different sensors was much higher (12.76% compared to 4.96% for acid cleaning). With the aim of minimizing variability between electrodes, acid cleaning was deemed the best approach here. No statistically significant difference was observed in terms of the parameters of interest across the different acid molarities tested for sulfuric acid treatment, thus the lowest molarity (0.1 M) was selected for the rest of the work.

### Fluidic chambers can be designed and manufactured for easy integration with commercial electrodes

**Figure 2:**
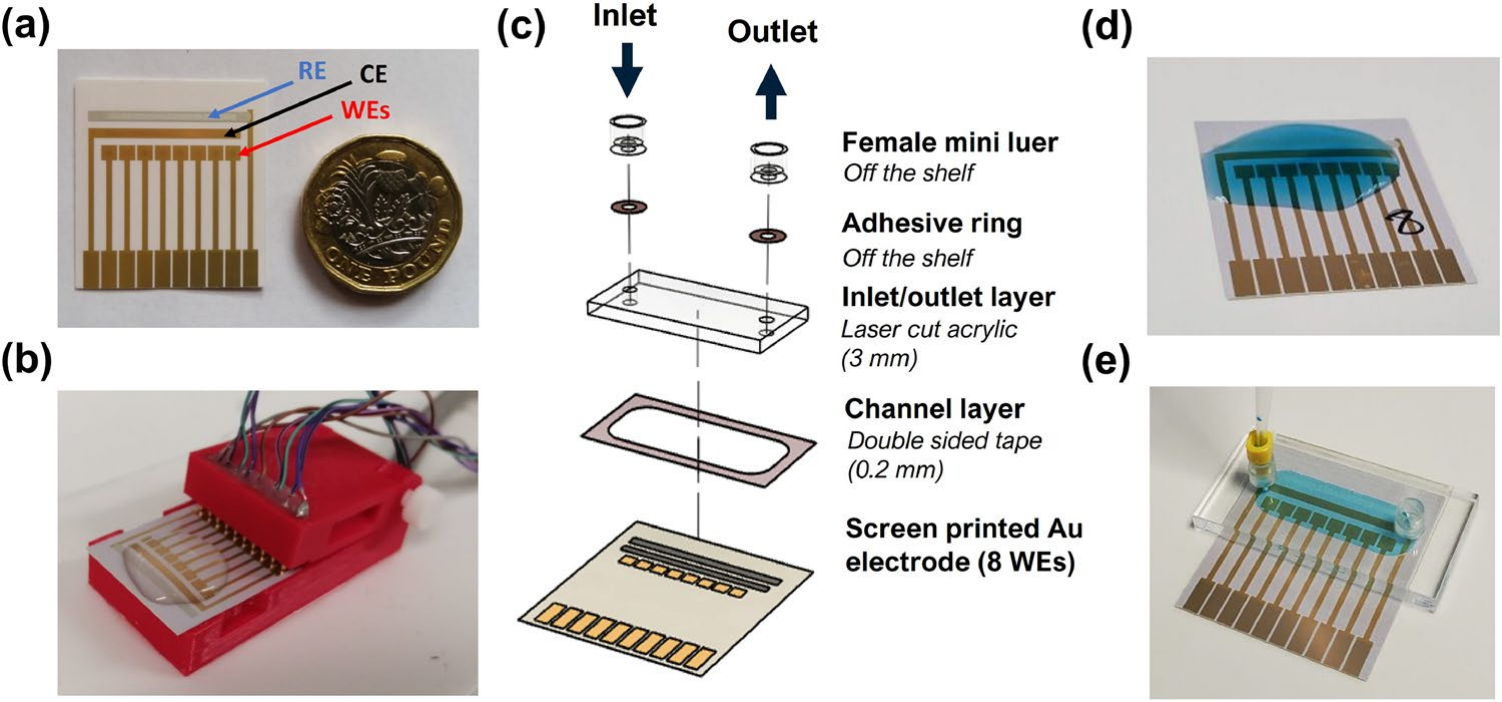
(**a**) The commercial screen-printed electrode platform used in these experiments features 8 individual working electrodes (WEs) and a common Ag/AgCl pseudo reference (RE) and Au counter electrode (CE). (**b**) The typical experimental set-up with the electrode platform placed inside a 3D printed connector made in-house and the reagents drop-cast on top of the electrodes. (**c**) Proposed flow cell design consisting of a laser cut inlet/outlet layer made from PMMA, channel layer made from double sided polyester tape cut to size using a Cricut electronic cutter and off-the-shelf mini luer fluidic adaptors attached to the PMMA using circular adhesive rings. (**d**) Before and (**e**) after flow cell attachment to the electrode platform.

The benefits of minimizing sample volumes during electrochemical measurements have already been demonstrated^19,40^. While this is traditionally done via microfluidic integration, most fluidic manufacturing processes are challenging to couple with commercial (plastic) electrodes such as the ones used in this work (Fig. 2a,b). In Fig. 2c, we introduce the flow cell—a fluidic chamber that consists of only 2 layers: an inlet/outlet layer with 2 holes for the inlet and outlet adaptors made of laser cut 3 mm acrylic, and a channel layer made of 230 µm thick double-sided tape, cut using an electronic cutter (cf Materials and Methods). The two layers are designed so that the working, counter and reference electrodes are all covered, while the layers are easily bondable (via the double side tape) to plastic electrodes. The use of laser and electronic cutting enables rapid alteration of the fluidic design to fit different electrode formats available off-the-shelf. The top layer is designed to work with off the shelf connectors, here pipette to mini-luer adaptors for sample addition. This allows fluid manipulation in a pump-free manner, substantially cutting down the cost and complexity of the system while retaining the advantages of having a microfluidic device (lower reagent volumes, minimal contamination, and reduced evaporation of the liquid). The flow cell can be used in different ways by changing the connectors *e.g.*, it can be connected to a syringe pump for automated sample/reagents injection. The bonding strategy proposed can withstand flow rates of dozens ml/min without leaking, although when working with sensitive reagents such as antibodies, flow rates at the lower end of the working range may be preferable (< 1 ml/min). The total cost for one flow cell is only  £2.7, cost that can be further lowered if other fluidic connectors are used (here off-the-shelf connectors are used as they are easily accessible and versatile for non-experts) or directly embedded in the platform design (see Table S2 for a breakdown of the materials cost).

Without flow cell, the minimum volume needed to cover all the electrodes is 400 µl (Fig. 2d); with the flow cell this volume can be reduced to 40 µl, or a 10-fold reduction (Fig. 2e). To encourage user adoption, we investigated here addition of the flow cell only at the final step of the preparation procedure, where the enzymatic reaction to detect IL-6 gives rise to a colorimetric signal measurable by chronoamperometry. This decision is motivated by the fact that preparation procedures for electrochemical sensing can be lengthy (typical sandwich ELISA biosensor preparation and measurement protocols can take around 7 h from start to finish) and addition of the flow cell earlier can be seen as disruptive for established workflows. Once the individual components of the flow cell are fabricated, assembly only takes $\sim$1 min per flow cell (Video S1).

### Spontaneous reaction between the pseudo-reference and TMB can be observed in micro-environments

3,3’,5,5’-tetramethylbenzidine (TMB) is a chromogenic substrate typically used in ELISA assays for signal amplification following binding of the target analyte. In the presence of enzymes such as horseradish peroxidase (HRP) and hydrogen peroxide, the reduced form of TMB (clear) becomes oxidized, which changes its colour to blue. The more target that is bound by the capture antibody, the more enzymes will be present at the end of sensor functionalization process (before addition of TMB), and consequently a stronger color change will be developed in the medium. However, this reaction can in some cases be modulated through the presence of certain catalysts *e.g.*, metallic silver ($Ag^+$), which can either enhance^41,42^, inhibit^43^, or even reverse^44^ this reaction in a pH-dependent manner.

**Figure 3:**
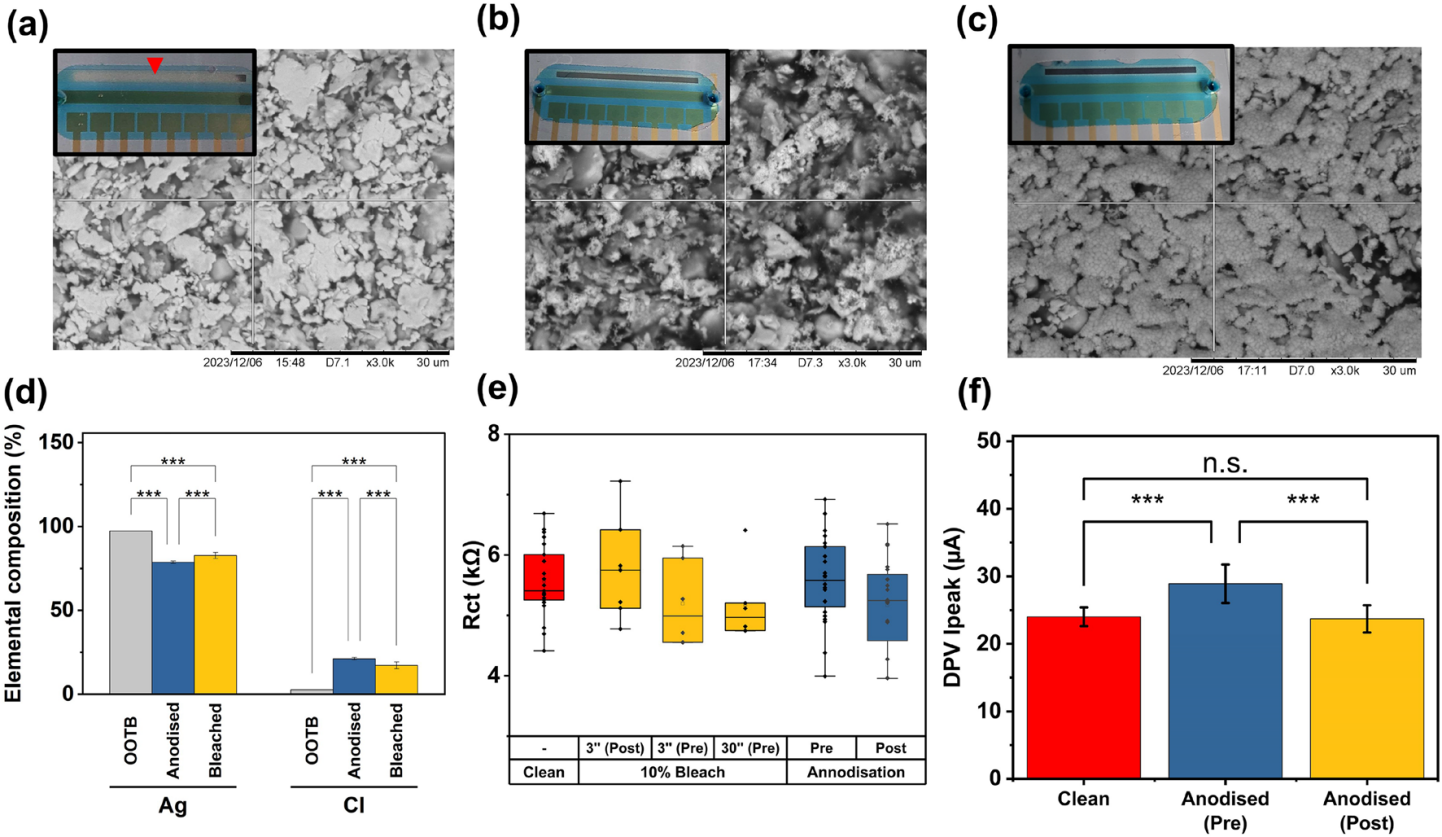
(**a**) Spontaneous reaction taking place between the Ag/AgCl pseudo-reference electrode and 3,3’,5,5’-tetramethylbenzidine (TMB) locally reducing the oxTMB produced by the HRP-TMB reaction taking place on the detection surface superimposed on SEM scan of the reference electrode surface. (**b**) The Ag/AgCl reference after being submerged in 10% v/v bleach for 30 min exposed to a solution of oxTMB—the cross-reactivity is reduced but not eliminated and the surface appears damaged by the bleach. (**c**) The Ag/AgCl reference after anodization in 1 M NaCl exposed to a solution of oxTMB—the cross reactivity with TMB is minimized and the integrity of the Ag crystals appears to be maintained. (**d**) Elemental composition analysis of Ag/AgCl reference electrode surface and the impact of anodization and bleaching treatments by energy dispersive X-ray Spectroscopy (bars and SD are based on 10 different measurements taken in different locations across the reference electrode surface). (**e**) The effect of bleaching and anodization treatment and interplay with sulfuric acid cleaning (pre or post Ag/AgCl treatment) on the charge transfer resistance of the Au surface. Each box delineates the interquartile range (IQR), the straight line denotes the median, the hollow square represents the mean and whiskers denote the range within 1.5xIQR. (**f**) The difference between the peak current of the differential pulse voltammogram when the Ag/AgCl electrode is anodized before (pre) as opposed to after (post) sulfuric acid cleaning highlighting that for best results cleaning must be carried out before anodization. Statistical significance is indicated by * where *** denotes $p<$=0.001. n.s. = non-significant.

During initial experiments, the silver pseudo-reference electrode was observed to spontaneously reduce the TMB substrate, changing its color back to clear, acting in a peroxidase-like fashion (Fig. 3a inset). This reaction took place quickly (<1 min), without any external voltage, and the volume affected accounted for approx. 10% of the total volume within the flow cell. Consequently, the amount of oxidized TMB available for detection was decreased, which could lead to an underestimation of target presence. To resolve this issue, 2 possible solutions were investigated: exposure of the Ag/AgCl pseudo-reference to a solution of 10% v/v sodium hypochlorite (NaClO, more commonly known as bleach) and anodization in a 1 M solution of sodium chloride (NaCl) (Fig. 3b,c insets). Both methods aim to increase the amount of AgCl (non-reactive) relative to Ag on the surface of the reference electrode through chlorination, which was indeed the case as shown in Fig. 3 d where the percentage of elemental Cl is substantially increased following both treatments. Anodization appeared to deposit more Cl on the electrode surface and resulted in a more uniform surface coverage compared to bleaching (Fig. 3b,c). In fact, with bleaching, the original Ag/AgCl crystals became difficult to distinguish on the scanning electron microscope scan compared to the out of the box (OOTB) condition (Fig. 3a main figure) hinting that this technique may damage the surface of the reference electrode. It was found that, out of all the conditions investigated, anodization was the only modification capable of stopping the pseudo reference interaction, whereas bleaching only delayed it for a short amount of time ($\sim$5–10 min)—the later could be due to the presence of large gaps present in the surface following treatment leaving a large portion of Ag still exposed and able to react with the substrate. None of the modifications statistically significantly affected the electrochemical charge transfer properties of the system (Fig. 3e), but as shown in Fig. 3f, anodizing after acid cleaning seemed to alter the variability of the sensors to a lesser extent (compared to doing it the other way around) and therefore this method was taken forward.

### The flow cell significantly enhances electrochemical detection of IL-6 in samples

**Figure 4:**
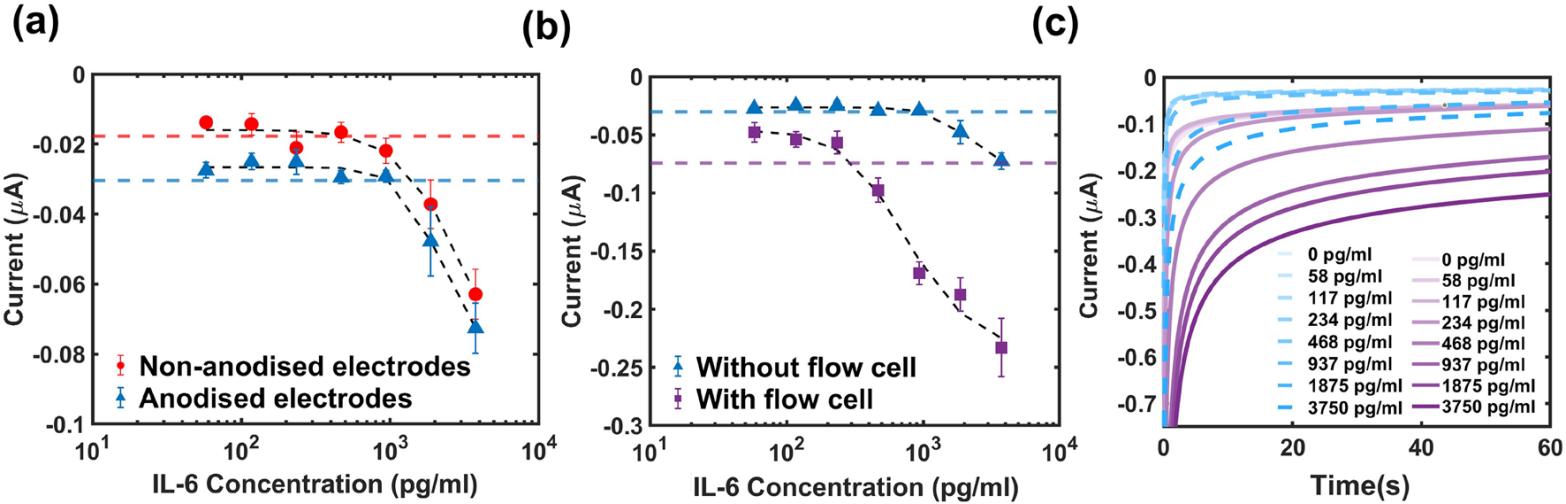
(**a**) The difference between the dose-dependent responses of the electrochemical immunosensor to IL-6 spiked in PBS for unmodified (red) and anodized (blue) electrodes during fully manual preparation (no flow cell present). Top (red) and bottom (blue) dashed lines represent the average blank reading—3 SDs for unmodified (n = 8) and anodized electrodes respectively (n = 6). (**b**) The effect of adding the flow cell at the final step of the sensor preparation procedure compared to fully manual preparation and testing (IL-6 spiked in PBS). Both responses are with anodized electrodes. Each marker in (**a**) and (**b**) represents the average over all measurements +/− 1 SD and the top (blue) and bottom (purple) dashed lines represent the average blank reading—3 SDs for the ‘without’ and ‘with flow cell’ conditions respectively (n = 6). Further details on the blank means and +/− 3 SD interval can be found in Table S3. (**c**) Dose-depended transient current response to different concentrations of IL-6 spiked in PBS without (dashed, blue lines) and with the flow cell (purple, solid lines) showing increase in sensitivity upon addition of the flow cell (single measurement per line). Both datasets were acquired with anodized electrodes.

Next, we isolated the effects of anodizing the silver pseudo-reference and adding the flow cell at the end of the sensor preparation procedure on the performance of the IL-6 electrochemical sandwich immunosensor in a non-complex sample (PBS buffer). It was noticed that when the substrate was drop cast onto the electrodes, anodizing the silver electrode shifted the current dose response curve downwards by around 19.7 nA (Fig. 4a). This can be attributed to a potential shift of   50 mV in the TMB oxidation peak registered by the electrode (Fig. S4), which was not corrected in the step voltage applied during the chronoamperometry measurement. Since the overall dose-response in the case when the substrate was dropcast onto the electrode (as opposed to being introduced to the sensing surface via the flow cell) seemed to be unaffected by anodisation except for the 19.7 nA current offset, for further experiments this modification was only applied when working with the flow cell.

Adding the flow cell not only lowered the limit of detection (LoD) from 1875 to 468 pg/ml (Fig. 4b), but it also improved the sensitivity of the sensor around 5.5-fold over the higher concentration range (468–3750 pg/ml). This increase in sensitivity is also evident in the time dependent current transients showing a much wider response range compared to before (Fig. 4c). This boost in performance was attributed to the spatial confinement of the oxidized TMB in the region directly above the detection surface, which limits diffusion of electroactive species into the bulk solution where they are harder to detect.

**Figure 5:**
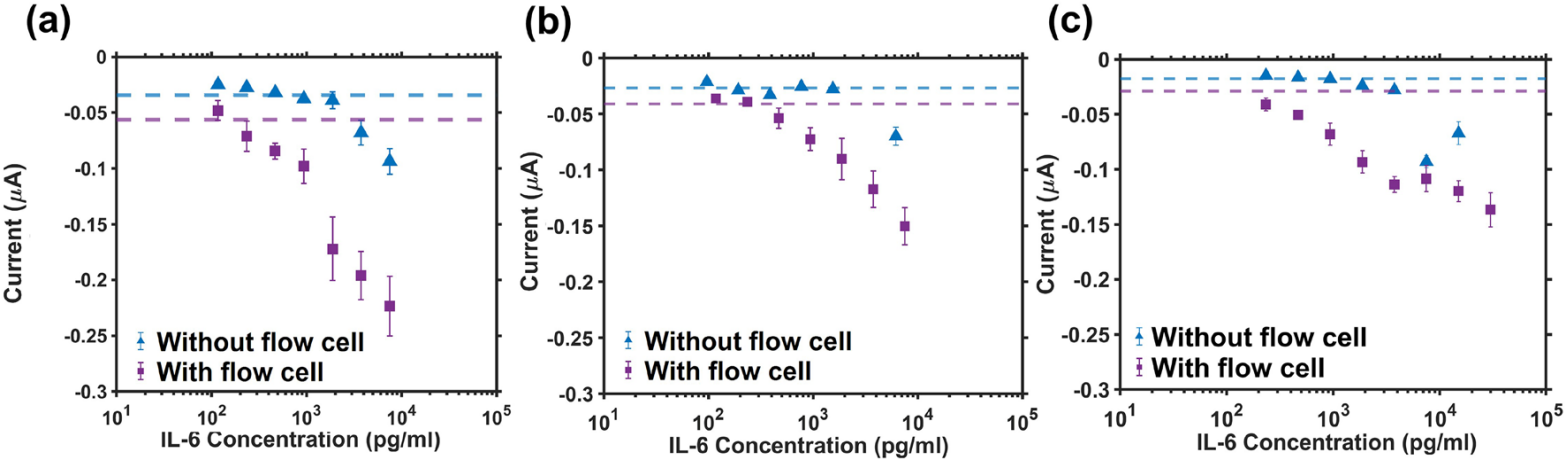
(**a**) Dose-dependent response of the electrochemical immunosensor to IL-6 spiked in 0.2 µm filtered human serum (10% v/v) with and without the flow cell present at the final detection step showing a large increase in sensitivity following the addition of the flow cell. (**b**) Dose-dependent response of the electrochemical immunosensor to IL-6 spiked in 10% unfiltered human serum with and without the flow cell present at the final detection step showing a substantial increase in sensitivity and the limit of detection following the addition of the flow cell. (**c**) Dose-dependent response of the electrochemical immunosensor to IL-6 spiked in neat unprocessed human plasma before and after integrating the flow cell into the preparation protocol. Each marker represents the average over 8 measurements +/− 1 SD and the horizontal dashed lines represent the average blank reading (0 pg/ml)—3 SDs (n = 8). Further details on the blank means and +/− 3 SD interval can be found in Table S3.

A dramatic increase in sensitivity (x5.8) was noted with the flow cell for IL-6 spiked in 10% human serum (0.2 µm filtered—Fig. 5a). An additional control experiment was also carried out at confirming the importance of the primary antibody within the detection scheme and assay specificity to IL-6 (Fig. S5). However, in our previous work we have demonstrated more extensively the specificity and selectivity of these reagents for their target and their suitability for this type of application^45,46^.

Unprocessed human serum and neat human plasma (Fig. 5b,c) had a very poor response when the flow cell was not present, with only high concentrations (>3 ng/ml) eliciting a response. Upon addition of the flow cell, the LoD was considerably lowered, and the dose-dependent curve could be clearly distinguished. The LoDs of the biosensor in different media before and after the addition of the flow cell are summarized in Table 1 below. The dose-response behaviors of the sensor with the flow cell are also compared in Fig. S6. The lowest limit of detection was achieved when testing the sensor in neat human plasma, whereas the highest sensitivity over the higher working range (>1 ng/ml) was in PBS and filtered dilute serum. This improvement in the LoD in the most complex samples tested could be attributed to the presence of molecular factors that are critical to antibody function and stability (e.g. albumins, protease inhibitors)^47^ that are absent in ‘cleaner’ media such as PBS. This result also showcases that the additional filtration step may actually remove some of these critical compounds leading to a slightly higher LoD. However, this mechanism may vary depending on the antibody-target combination. In future work, we plan on exploring the effect of the flow cell on different assays to further elucidate this. Another interesting effect was noted when comparing the behavior of the sensors modified with the flow cell over the ultrahigh working range with and without anodisation of the reference electrode—Fig. S7. It was hypothesised that more substantial effects will likely take place at these higher concentrations as more of the TMB will become oxidised and consequently diffuse towards the reference electrode. What we saw is that for non-anodised electrodes, a Hook effect appears to be taking place around 10 ng/ml whereas that appears to be damped in the anodized configuration.

**Table 1:** Limits of detection (defined as the first data point falling outside of the 99% confidence interval around the blank reading) before and after flow cell addition - a comparison between different sample types.

Sample tested	LoD (pg/ml)	
	With flow cell	Without flow cell
PBS	468	937
10% (v/v) Human Serum (0.2 µm filtered)	234	468
10% (v/v) Human Serum (un-filtered)	468	3080
Neat Human Plasma	<234	1875

## Conclusion

This paper presents a low-cost flow cell design that can be easily fabricated using consumer grade materials (PMMA sheets, double sided adhesives, and off-the-shelf fluidic adaptors) and methods (laser and electronic cutting). The fabrication method proposed is accessible to researchers from a non-microfluidic or engineering background, allows for rapid and accurate prototyping cycles and, once a suitable geometry has been identified, the production of large numbers of fluidic cells in a short time frame which can be stored until ready for use. We validated this flow cell using a commercial gold electrode platform and a sandwich electrochemical immunoassay format for detecting IL-6, an inflammatory cytokine of interest across a variety of different pathologies. First, we determined the optimal electrode cleaning protocol to ensure that the Au detection surface was devoid of contaminants that could interfere with the electron transfer processes. We found that electrochemical voltage cycling in 0.1 M sulfuric acid for 10 cycles yielded sensors with substantially lower overall variability compared to $O_2$ plasma etching. Once we started using the flow cell with the immunoassay detection format, we observed a cross-reactivity between the Ag/AgCl pseudo reference and the ELISA substrate (TMB), which was decreasing the amount of oxidized TMB produced by the target-antibody immunocomplexes immobilized on the electrode surface. This effect was, in the absence of the flow cell, impossible to discern visually through the large volume of bulk fluid that is needed to cover all electrodes during testing. With the aid of the flow cell, we also found that this effect could be eliminated with minimal impact to the sensing quality by anodizing the reference electrode in a 1 M NaCl solution for 1 min at 1 V DC.

Finally, we tested the sensor in 4 different types of media: PBS, 10% v/v 0.2 micro filtered human serum, unfiltered 10% v/v human serum and neat human plasma. To facilitate user adoption and minimize disruption to already established workflows, the potential of the flow cell is demonstrated here at the final step of the electrochemical immunosensor preparation protocol, when the signal amplification reaction takes place. We showed that by confining the product of the enzymatic reaction to the vicinity of the detection surface, a substantial increase in the sensitivity of the sensor and decrease in the limit of detection could be obtained in both spiked buffer and complex media such as human plasma. We envision that in the future, the flow cell will become an invaluable accessory in the electrochemical biosensor researcher’s toolkit, allowing faster and more sensitive sensors to be developed at a low price point and eventually facilitate translation of these sensors into the clinical environment.

## Methods

### Chemicals and electrodes

Sulfuric acid (98%), potassium ferricyanide (99+%, for analysis), potassium ferrocyanide trihydrate (99+%, for analysis), human serum (H4522) and PBS tablets were all purchased from Merck. Ultrapure water (18.2 M$\Omega$.cm) was obtained from an Elga Purelab Chorus water purification system. Primary and secondary IL-6 antibodies, IL-6 standard and x40 horseradish peroxidase concentrate (HRP) were all purchased from R &D Systems Inc (Minneapolis) as part of a Human IL-6 DuoSet ELISA kit (DY206). Reagent diluent x10 (1% BSA in PBS, pH 7.2-7.4, 0.2 µm filtered, DY008B), TMB substrate (DY999B) and PBS plate-coating buffer (DY006) were also purchased from the same manufacturer. The Au electrodes used in these experiments were manufactured by FlexMedical Solutions (Livingston, UK) and feature 8 working electrodes and a common reference and counter electrode on each chip. The Ag/AgCl (CHI111) and Au (CHI101) macroelectrodes used for the anodization experiments were purchased from IJ Cambria Scientific Ltd. The 0.2 $\mu$m membrane syringe filters were from Whatman (cat. number 6780-2502).

### Cleaning experiments

In these series of experiments, two common cleaning methods were compared to determine the optimal protocol for removing contaminants from the Au electrode surface and facilitate sensing: sulfuric acid voltage cycling and oxygen plasma etching. Three different acid strengths were investigated including 0.1, 0.2 and 0.5 M. Before use, the electrodes were rinsed with ultrapure water and dried under stream of argon. Following this, 600 $\mu$l of acid was drop cast onto the electrode platform ensuring that all electrodes were adequately covered. The potential was then scanned on each working electrode between $-0.5$ and 1.6 V using a scan rate of 0.1 V/s for 10 cycles using a PalmSens4 potentiostat equipped with a MUX-R2 multiplexer unit from PalmSens. The software PSTrace 5.9 was used to control the potentiostat. After cleaning, the electrodes would again be rinsed with ultrapure water and dried with argon making them ready for use. For plasma cleaning, we used a Zepto plasma asher (200 W) connected to an oxygen gas cylinder set to different time and power settings. 3 electrodes were placed inside the asher chamber during the cleaning process. To characterize the electrodes, we used a 2 mM solution of $Fe[CN]_{6}^{3-} + Fe[CN]_{6}^{4-}$ (Ferri-Ferro Cyanide) redox couple in 1xPBS (made from tablets) and interrogated the system using cyclic voltammetry, differential pulse voltammetry and impedance spectroscopy. For the cyclic voltammetry measurement, we scanned the potential between $-0.4$ and 0.5 V in 0.01 V steps using a scan rate of 10 mV/s. The same voltage window, step size and scan rate were used for the DPV characterization. EIS measurements were performed against the open circuit potential of the cell, over the range 0.1–100,000 Hz (83 frequencies) with an AC potential of 10 mV. To extract the Rct values, data was fitted in PsTrace 5.9. using Randles equivalent circuit with a constant phase element (CPE) instead of an ideal capacitor (Fig. 1 a inset) to account for non-ideal sensor behaviour. The Rct values for individual electrodes were averaged and the error was calculated as 1 standard deviation from the mean.

### Fabrication and assembly of the flow cell

The flow cell was constructed using 2 distinct layers: a inlet/outlet layer made from 3 mm laser cut acrylic with two holes (1.3 mm diameter) and a channel layer made from double sided tape cut using a Cricut$\circledR$ Maker 3 to delineate a channel exposing all electrodes. The PMMA sheet for the inlet/outlet layer was purchased from Stockline Plastics Ltd (Glasgow, UK) and the 230-$\mu$m thick double sided polyester tape was from RS Components (RS Stock No.: 468-380). The geometry for the inlet/outlet layer was created in Serif DrawPlus X8, which can upload the design directly to the laser cutter. The geometry for the channel layer was created in the Cricut Design Space App which is available with all Cricut cutters. To cut the inlets layer we used an Epilog Mini 50-Watt laser cutter set to 30% speed, 100% power, 5000 Hz frequency. For the channel layer, since the tape was on a roll format, it had to be unraveled and placed on a temporary release silicone film before being placed on a Strong Grip Cricut$\circledR$ cutting mat (12” $\times$ 12”). Masking tape was also added at the edges to ensure that the silicone film remains in place during cutting. The cutting settings used were the standard Cricut$\circledR$ ones for double-sided adhesive which resulted in a clean cut of the double-sided tape. For introducing the reagent into the flow cell, we used mini female luer adaptors from the Microfluidic ChipShop (Fluidic 631) and a pipette to mini luer adaptor from the same supplier (Fluidic 391). The adaptors were attached to the flow cell via adhesive rings cut to size (Fluidic 699).

### IL-6 sensor preparation procedure and interrogation

After cleaning with 0.1 M sulfuric acid, some of the silver pseudo reference electrodes were anodized by submerging in a 1 M NaCl solution and applying a 1 V potential for 60 s with a 0.1 s time interval which changed them to a dark brown color. This was to prevent the spontaneous reaction between the Ag/AgCl pseudo reference and the TMB substrate observed during preliminary experiments. To begin the sensor preparation, 2 $\mu$l of 100 $\mu$g/ml primary IL-6 antibody diluted in ELISA plate coating PBS buffer was pipetted on each working electrode. The electrodes were then incubated overnight at 4 $^{\circ }$C inside a humidity chamber. The next day, the electrodes were gently washed 3 times with 1 ml of 1xPBS to remove any unbound antibodies. This wash step was repeated after each incubation step. Next, the electrodes were incubated with reagent diluent (1% BSA in PBS) for 1 hour at room temperature to minimize any non-specific interactions with the detection surface. Standard dilutions of the target were then prepared in the medium of interest (PBS, filtered or unfiltered dilute serum and plasma), placed on the individual electrodes and incubated for 1 h at room temperature. Il-6 secondary antibodies were then prepared in 1x reagent diluent at double the concentration stated in the certificate of analysis (in our case this was 100 nM, but this may change between different batches) and incubated for a further hour. HRP was then diluted to 1x in reagent diluent and placed on the electrodes for 20 minutes, washed off and then 400 $\mu$l of TMB substrate was added covering all electrodes (including the counter and the reference) and incubated for 20 more minutes. If the flow cell was used, the release liner would be peeled off the bottom of the channel layer and the flow cell manually installed using the edge of the electrode platform and installation guide lines laser engraved onto the top side of the flow cell to ensure proper alignment (Video S2). The flow cell was then filled with 40 $\mu$l of TMB using a pipette and a pipette to mini luer adaptor (Fluidic 391). The last 2 incubations steps were performed in the dark (electrode storage boxes were wrapped in tinfoil). After the final incubation step, the presence of target was indicated by the production of a blue product (oxidised form of TMB) which was measured using chronoamperometry by applying $-0.2$ V for 60 s with a 0.1 s time interval. The stable value of the current after 60 s was then extracted from PSTrace and plotted in MATLAB R2022a.

### Extraction of plasma from blood samples

The study was approved by the ethics committee of the University of Strathclyde (UEC23/05).Whole human blood was acquired from Research Donors (https://researchdonors.co.uk) in a 10 ml lavender topped K2EDTA blood collection tube. To extract the plasma, the blood was centrifuged for 5 min at 4100$\times g$ and then the supernatant was extracted into a clean 15 ml Eppendorf tube and used the same day.

### Scanning electron microscopy (SEM) and energy dispersive X-ray spectroscopy (EDS) analysis

Electron microscope scans were acquired using a Hitachi TM-1000 tabletop microscope and elemental analysis was conducted using an Oxford EDS unit connected to the main SEM unit. The microscope and EDS unit were connected to a laptop with the TM-1000 and SwiftED EDS data acquisition software installed. The acquisition time for the EDS data was 8 s and all measurements were carried out with an n = 10, taken at different locations along the sample.

### Data fitting and statistical analysis

The peaks in the voltammetry data was extracted using the PSTrace 5.9 software and the impedance spectra was modeled using a standard Randles equivalent circuit with a constant phase element instead of an ideal capacitor and fitted in PSTrace using the Levenberg–Marquardt algorithm to extract the equivalent circuit parameters (*e.g.*, Rct). The chronoamperometry dose response curves were fitted using a 4-parameter logic regression model by least square fitting. The limit of detection was determined as the first experimental point outside of the ±3 standard deviation zone around the blank value (0 pg/ml IL-6). Further details on the means and standard deviations of all blank readings presented in this work can be found in Table S3. Graphics were produced in Origin 2022 graphing software and MATLAB R2022a.

### Supplementary Information

Supplementary Information.

Supplementary Information.

Supplementary Information.

Supplementary Information.

## Data Availability

All data generated or analysed during this study are included in this published article and its supplementary information files.
