# Supplementary material for: A plug-and-play, easy-to-manufacture fluidic accessory to significantly enhance the sensitivity of electrochemical immunoassays
Source: Sci Rep. 2024 Jun 19;14:14154. doi: 10.1038/s41598-024-64852-5 (PMC11187161; doi:10.1038/s41598-024-64852-5)
Supplement: Supplementary file 1 — Supplementary Information. [file 41598_2024_64852_MOESM1_ESM.docx]

Supporting Information

A plug-and-play, easy-to-manufacture fluidic accessory to significantly enhance the sensitivity of electrochemical immunoassays

Alexandra Dobrea*^1,2^, Nicole Hall^1^, Stuart Milne^1,3^, Damion K Corrigan^3^, Melanie Jimenez^1^

^1^Department of Biomedical Engineering, University of Strathclyde, Glasgow, UK.

^2^James Watt School of Engineering, University of Glasgow, Glasgow, UK.

^3^Department of Pure and Applied Chemistry, University of Strathclyde, Glasgow, UK.

*Corresponding author: [alexandra.dobrea@strath.ac.uk](mailto:alexandra.dobrea@strath.ac.uk)

Table S1. Coefficients of variability across the different conditions investigated for plasma etching (different power and exposure time settings) and sulfuric acid voltage cycling in sulfuric acids of various molarities (0.1-0.5 M) compared against out of the box (OOTB) electrodes. Values were calculated across 3 different electrode platforms for an n=24 for each condition. NT=not tested


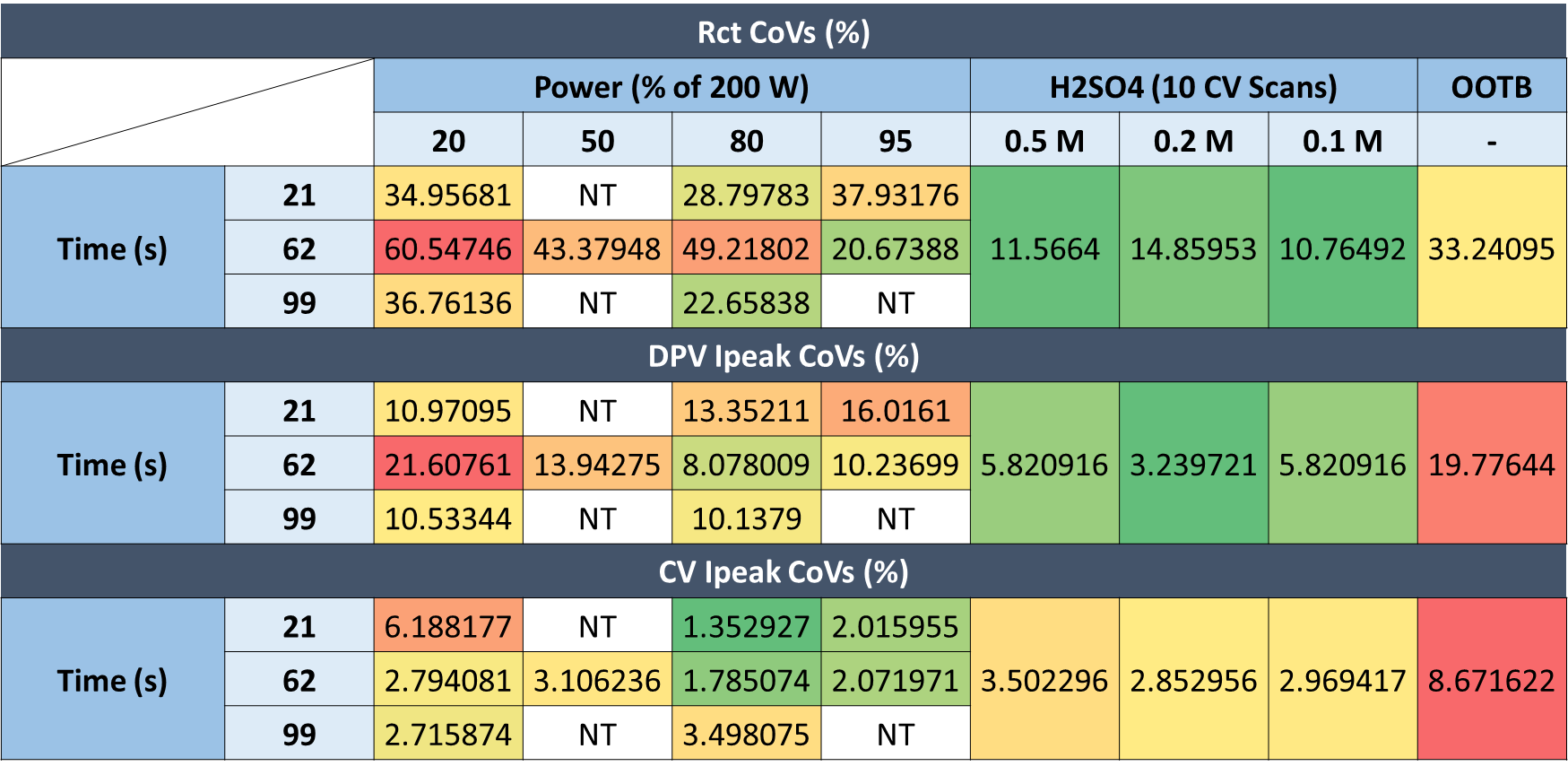

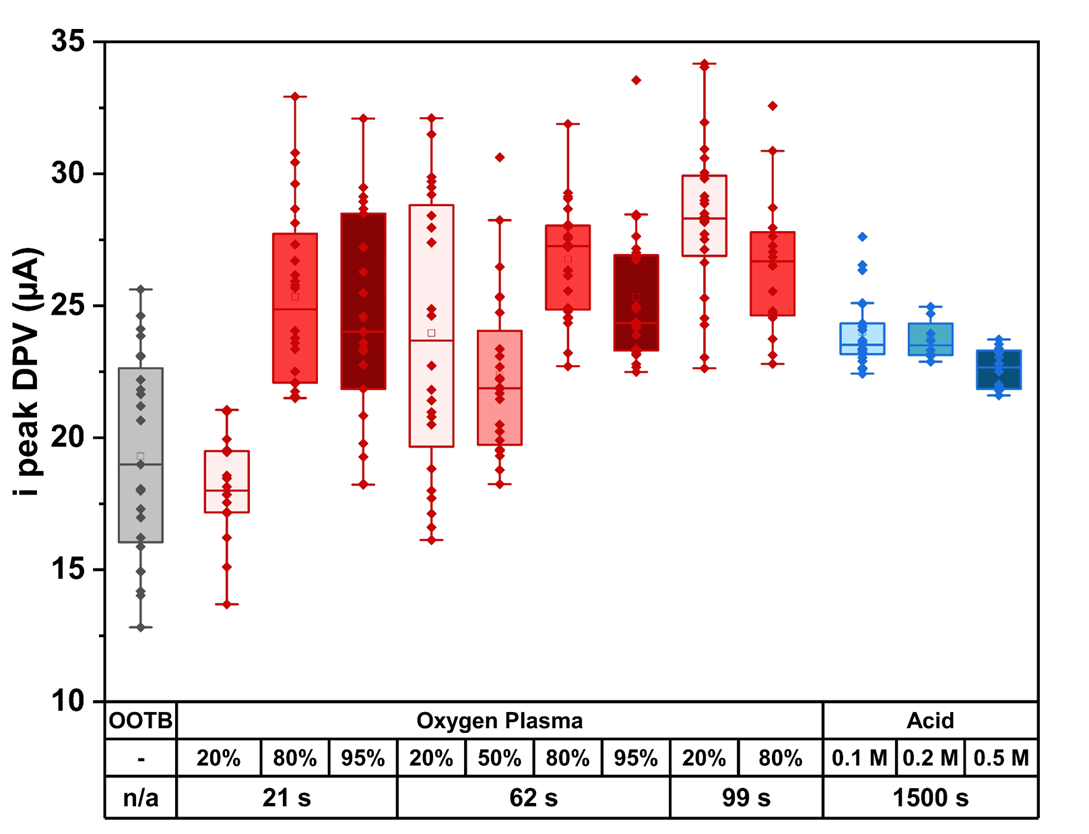


Figure S2. Comparison of the effect of oxygen plasma and sulfuric acid voltage cycling on the peak current of the differential pulse voltammogram. Datapoints represent 3 different screen-printed electrode platforms with 8 working electrodes each for an n=24 per tested condition. Each box delineates the interquartile range (IQR), the straight line denotes the median, the hollow square represents the mean and whiskers denote the range within 1.5xIQR. OOTB=out of the box.

Table S2. Material costs for fabricating one flow cell. Prices are from Stockline Plastics Ltd. (Glasgow, UK), RS Components (Glasgow, UK) and Microfluidic ChipShop (Germany).


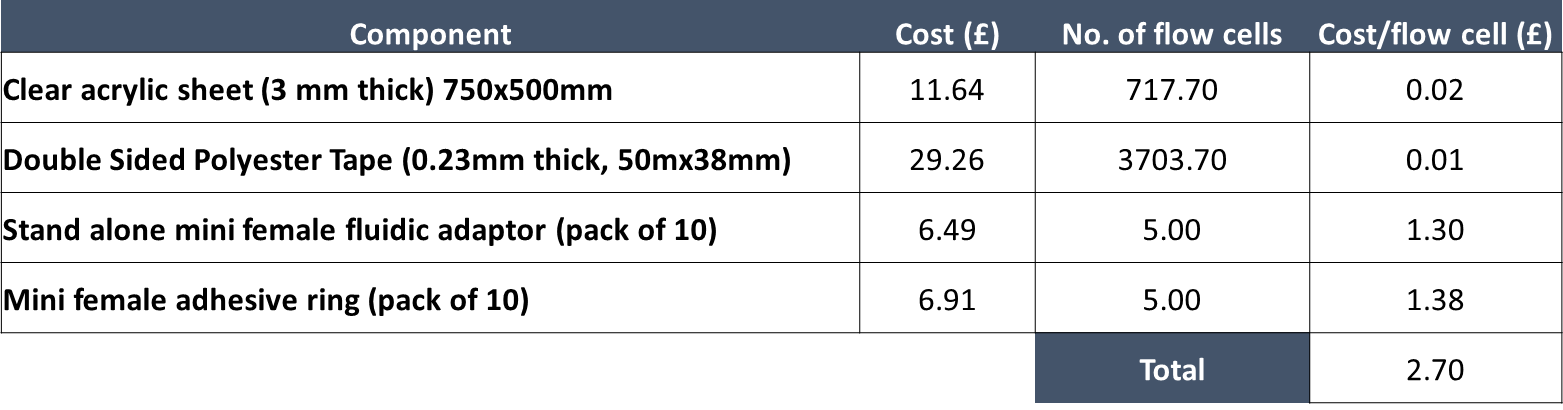


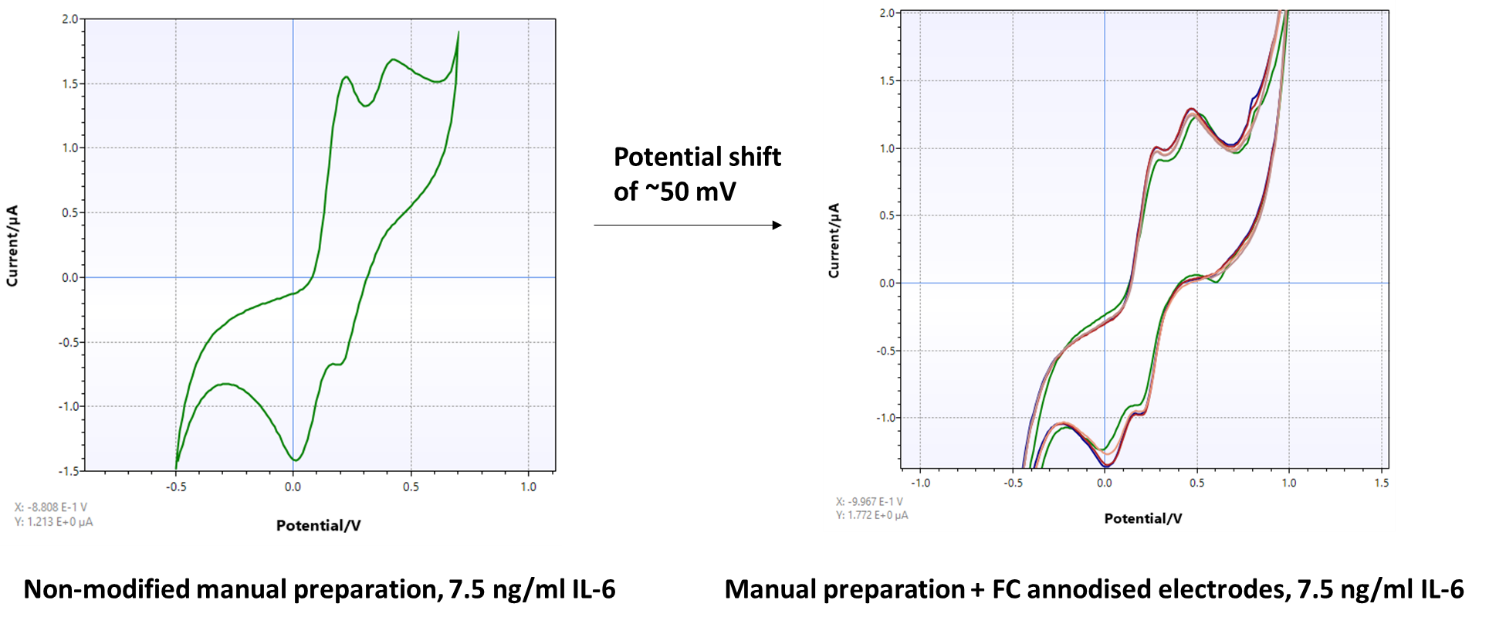


Figure S4. Potential shift observed in the characteristic TMB cyclic voltammogram induced by the anodization of the Ag/AgCl reference electrode. (Left) Non modified reference electrode (Right) Anodised reference electrode. Data acquired in PSTrace 5.9.


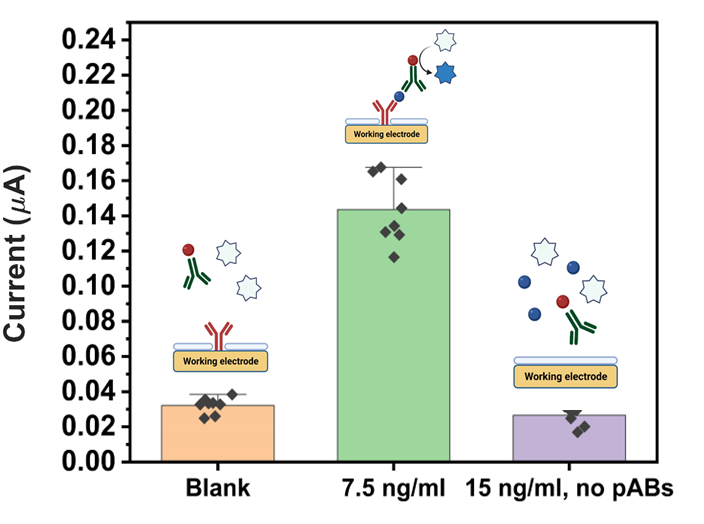


Figure S5. Control experiment carried out in 10% v/v unfiltered serum spiked with IL-6 confirming assay specificity to IL-6 (n=8)


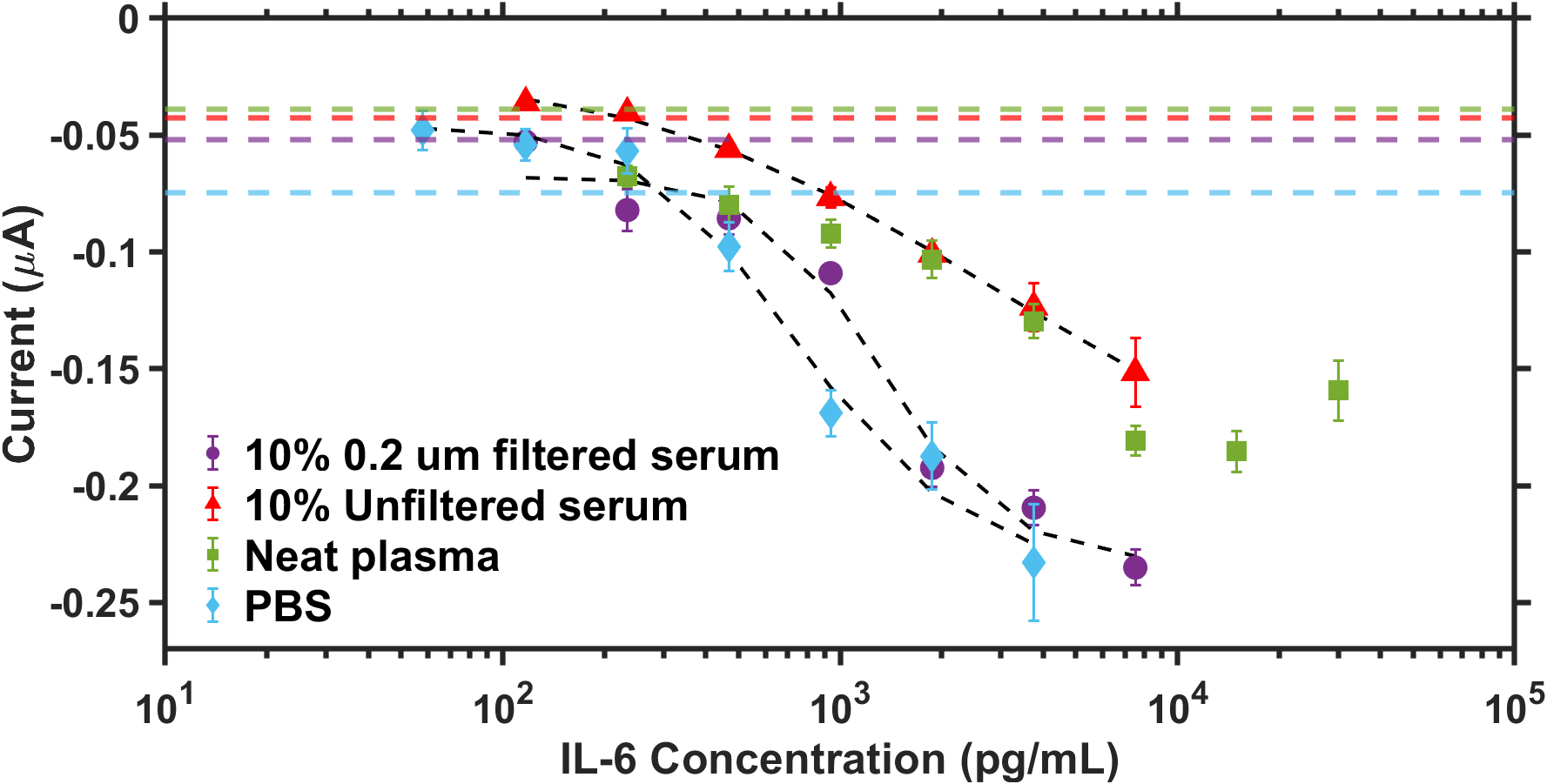


Figure S6. Dose response curves of the biosensor to different testing media: 10% 0.2 um filtered and unfiltered human serum, neat human plasma and PBS (n=8 per concentration point). Horizontal dashed lines represent the blank reading (0 pg/ml) – 3 SDs (n=8). Black dashed lines are the lines of best fit for each dataset (4 parameter logistic regression model).


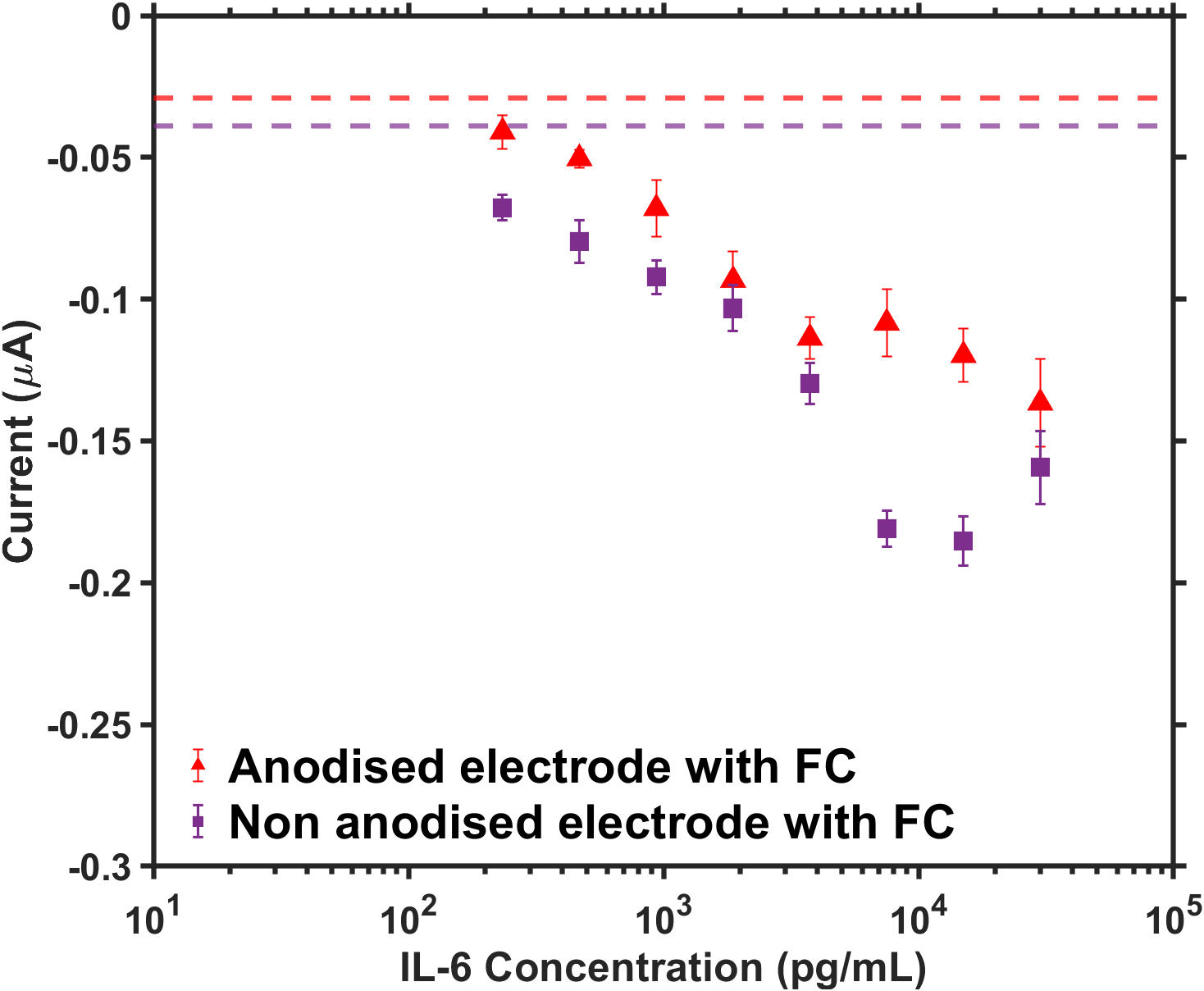


Figure S7. Side by side comparison between anodized and non-anodized electrodes with the flow cell attached and the effect on the dose-response to IL-6 suggesting a dampening of the Hook effect over the ultrahigh concentration range. Datapoints are average over 8 measurements (1 electrode platform/concentration point. Dashed lines represent the blank reading (0 pg/ml) – 3 SDs (n=8).

Table S3. Blank (0 pg/ml IL-6) readings displayed in the main paper Figures

| **Condition/Sample tested** | **Figure** | **Blank current value ± 3 SD (nA)** |
| --- | --- | --- |
| Non-anodised electrodes dose-response/PBS | 4a – red (top) line | -15.5 ± 2.2 |
| Anodised electrodes dose-response/PBS | 4a – bottom (blue) line | -26.3 ± 6.8 |
| Anodised electrodes dose-response (**without** **flow cell)**/PBS | 4b – top (blue) line (same as 4a bottom/blue line) | -26.3 ± 6.8 |
| Anodised electrodes dose-response (**with** **flow cell**)/PBS | 4b – bottom (purple) line | -62.0 ± 12.6 |
| Non-anodised electrodes **without flow cell**/10% (v/v) 0.2 µm filtered serum | 5a – top (blue) line | -26.1± 8.0 |
| Anodised electrodes **with flow cell**/10% (v/v) 0.2 µm filtered serum | 5a – bottom (purple) line | -36.3 ± 19.0 |
| Non-anodised electrodes **without flow cell**/10% (v/v) un-filtered serum | 5b – top (blue) line | -21.2 ± 5.7 |
| Anodised electrodes **with flow cell**/10% (v/v) un-filtered serum | 5b – bottom (purple) line | -32.2 ± 13.6 |
| Non-anodised electrodes **without flow cell**/neat plasma | 5c – top (blue) line | -14.7 ± 2.9 |
| Anodised electrodes **with flow cell**/neat plasma | 5c – bottom (purple) line | -20.8 ± 8.2 |
